# Early Lessons on the Importance of Lung Imaging in Novel Coronavirus Disease (COVID-19)

**DOI:** 10.4269/ajtmh.20-0225

**Published:** 2020-04-03

**Authors:** Arjen M. Dondorp, Marcus J. Schultz

**Affiliations:** 1 Mahidol–Oxford Tropical Medicine Research Unit (MORU), Mahidol University, Bangkok, Thailand;; 2 University Medical Centers Amsterdam, Location AMC, Amsterdam, The Netherlands;; 3 Nuffield Department of Medicine, University of Oxford, Oxford, United Kingdom

The novel coronavirus disease (COVID-19) pandemic is rapidly expanding across the world, with more than 60,000 new cases each day as of late March 2020. Healthcare workers are struggling to provide the best care for patients with proven or suspected COVID-19. Approaches for clinical care vary widely between countries and regions, and new insights are acquired rapidly. This includes the use of available imaging techniques.

In this issue of the *American Journal of Tropical Medicine and Hygiene*, Chaisith and others^1^ report a case of COVID-19 from Bangkok, Thailand, recovering with only supportive care.^1^ The case not only highlights the importance of early quarantining and repeated virologic testing of suspected COVID-19 patients but also demonstrates well that abnormalities on the chest X-ray can precede a positive severe acute respiratory syndrome coronavirus-2 reverse transcriptase polymerase chain reaction result from nasopharyngeal or throat swabs. Furthermore, Dr. Chaisith and others^1^ compared lung images from chest X-ray, chest computed tomography (CT) scan, and lung ultrasound (LUS) obtained later in the course of the disease.

In COVID-19, chest X-ray typically shows nonspecific multi-lobar infiltrates or pulmonary infiltration that can rapidly progress over 1–2 days. Chest CT scan findings in COVID-19 are more specific, including bilateral, multi-lobar, ground-glass opacification, which can become very extensive with disease progression, and multifocal consolidative opacities with surrounding spared tissue.^2^

Although these typical chest CT scan findings are highly informative, they are of limited value in resource-limited settings, where access to CT scanners is in general very limited. In addition, obtaining a chest CT scan is highly impractical once a patient is admitted to the intensive care unit for invasive ventilation because this requires dangerous and labor-intensive transportation to the CT scanner. Last but not least, there is a risk of contamination of the CT scanner and, thus, spread of the infection to other patients within the hospital.

The potential benefit of point-of-care LUS as a diagnostic tool in COVID-19 should be highlighted. Lung ultrasound is a well-established, noninvasive, rapid, repeatable, sensitive, bedside method to detect pulmonary pathology, including pneumothorax, pleural effusion, and pulmonary infiltrates or consolidations. Lung ultrasound has even been advocated as an alternative for chest X-ray and chest CT scan for diagnosing acute respiratory distress syndrome.^3–5^ Lung ultrasound is easy to perform and has almost no costs after the initial purchase of the ultrasound machine (around 10,000–15,000 USD). For poor patients in low- or middle-income countries, even a chest X-ray can be a considerable out-of-pocket expenditure if these costs are not covered by insurance or government funds. Also, chest X-rays in low-resource settings are often of poor quality, with a much lower sensitivity than LUS to detect pleural or parenchymal abnormalities.^6^

This information supports more extensive deployment of point-of-care LUS in COVID-19 patients, in particular in settings where resources are limited. The COVID-19 case presented by Chaisith describes LUS abnormalities observed at the 12th day of hospital admission. They did not show whether earlier COVID-19–related lung pathology is detectable by LUS, but this is probably the case. Whether LUS findings show COVID-19–specific features will need further exploration because this could provide a valuable diagnostic tool to distinguish COVID-19 from other pulmonary infections, and suggests the possibility of a severity score based on LUS findings. For example, the usual absence of pleural fluid in COVID-19 could be of diagnostic value because pleural fluid can be easily detected by LUS and is often present in patients with bacterial pneumonia and other conditions that result in respiratory distress, such as pulmonary congestion with heart failure.

Lung ultrasound could confirm on a larger scale whether the abnormalities observed on chest CT scan of multifocal opacities alternating with normal aerated lung represent a common lung pathology in COVID-19. This could have important practical consequences for ventilation strategies in individual patients. Focal areas of dense atelectasis, which are difficult or not to reopen, surrounded by remarkably compliant normal lung tissue, would imply that in patients needing invasive ventilation, high positive end-expiratory pressures (PEEP) may not be beneficial, and could even be harmful. Indeed, too high PEEP could cause overdistension and damage of ventilated unaffected lung areas, whereas the infected areas with atelectasis remain unventilated. This could lead to an increase in intrapulmonary shunt, worsening the already grave hypoxemia in severe COVID-19 disease. To further prove this concept, LUS could be used as a real-time imaging tool to evaluate the success or failure of attempts to reopen atelectatic lung tissue, for example, with high PEEP. Early experience from Amsterdam, the Netherlands, suggests that an emphasis on prone positioning and use of much lower PEEP in COVID-19 patients than in others with respiratory failure results in good patient outcomes (Schultz, personal communication).

In summary, LUS is a promising additional imaging tool in patients with COVID-19, in particular in resource-limited settings in low- and middle-income countries.
